# Dynamic Compressive Strength Tests of Corroded SFRC Exposed to Drying–Wetting Cycles with a 37 mm Diameter SHPB

**DOI:** 10.3390/ma14092267

**Published:** 2021-04-27

**Authors:** Hui Chen, Xiangqiang Zhou, Qiang Li, Rui He, Xin Huang

**Affiliations:** 1 Department of Architecture and Civil Engineering, Oujiang College, Wenzhou University, Wenzhou 325035, China; chenhui0306@wzu.edu.cn (H.C.); 20200365@wzu.edu.cn (X.Z.); 2 College of Architecture and Civil Engineering, Wenzhou University, Wenzhou 325035, China; 20461542031@stu.wzu.edu.cn; 3 College of Civil Engineering and Architecture, Zhejiang University of Water Resources and Electric Power, Hangzhou 310018, China; liq@zjweu.edu.cn; 4 College of Civil Engineering and Architecture, Zhejiang University of Technology, Hangzhou 310034, China

**Keywords:** dynamic compressive strength, corroded steel fiber-reinforced concrete (SFRC), drying–wetting cycles, split Hopkinson pressure bar (SHPB)

## Abstract

This study focuses on the dynamic compression performance of corroded steel fiber-reinforced concrete (SFRC) exposed to drying–wetting chloride cycles by a 37 mm diameter split Hopkinson pressure bar (SHPB) system. Three steel fiber contents (0.5%, 1.0%, 2.0%, by volume) were incorporated into concrete, and samples were subjected to drying–wetting cycles for different corrosion durations (30 days, 60 days, 90 days) after 28 days age. The sample damage mode, stress–strain curve and the dynamic compression performance of corroded SFRC were compared with plain concrete. Through the experimental data, strain-rate effect, fiber reinforcement effect and the corrosion duration influence on the impact compression property of SFRC were identified. The dynamic increase factor results of these samples were compared with the existing models in previous published literature. An empirical dynamic increase factor profile characterization model considering fiber content, corrosion duration and strain-rate is proposed.

## 1. Introduction

Concrete is the most widely used building material around the world due to its low cost, abundant availability of raw materials and strong compressive strength. However, the poor mechanical performance under high strain rate limits the application of ordinary concrete. Thus, a lot of studies have used fiber-reinforced concrete (i.e., polypropylene fiber [1], Barchip fiber [2], basalt fiber [3], etc.) to improve the mechanical properties of ordinary concrete. Steel fiber-reinforced concrete (SFRC) [4] has many engineering applications because of its better strength, ductility and impact loading resistance compared to ordinary concrete.

Over the past several decades, various studies have investigated the dynamic performance of SFRC under high strain rate. Lok and Xiao [5] studied SFRC panels exposed into air blast loading, and found that the failure pattern of the panels was deeply influenced by the fiber content. Marar et al. [6] used a drop weight method to study toughness energy and impact energy of SFRC, and a logarithmic relationship was found between the toughness energy and impact energy. Rong et al. [7] studied the dynamic performance of ultra-high-performance concrete (UHPC) incorporated with steel fiber. The results showed that with increasing steel fiber content, the impact resistance of UHPC increased. Hao and Hao [8] studied the effects of steel fiber content (0–1.5% by volume) and strain rate (50–200 s^−1^) on the dynamic compressive behavior of SFRC. The results showed that the compressive strength and elastic modulus exhibited increasing sensitivity to strain rate with increasing steel fiber content, and the energy absorption capacity had a strong dependency on the steel fiber volume fraction under high strain rate.

Due to the interconnected micropore structures [9], chloride ions can penetrate into concrete [10,11,12] and induce the corrosion of steel fiber. In particular, a drying and wetting cycle condition is always identified as the most unfavorable environmental condition for SFRC structures subjected to chloride-induced deterioration processes [13]. A few studies have been conducted to study the influence of steel fiber corrosion under the drying and wetting cycle condition and its influence on the mechanical performance of SFRC. Granju and Balouch [14] studied the corrosion of steel fiber in a cracked section of SFRC and surprisingly found that the static flexural strength of cracked SFRC samples exposed for 1 year to marine saline fog was increased, which is consistent with other researchers’ findings [15]. Marcos-Meson et al. [16] studied the deterioration phenomena of cracked SFRC exposed to wet–dry cycles of chlorides, and a conceptual deterioration model was developed, describing the deterioration and recovery mechanisms that alter the long-term mechanical performance of the cracked composite under wetting–drying conditions.

Several experimental methods can be used to study the dynamic performance of concrete (i.e., drop weight [6], air blast [5] and split Hopkinson pressure bar (SHPB) [17]). The SHPB is frequently applied to understand the variation in the strain rate and the compressive strength under different impact velocities. The SHPB was first developed by Hopkinson in 1914 [18] and revolutionary improvements were made by Kolsky in 1949 [19]. There are limited studies focused on the dynamic compression performance of SFRC by the SHPB. Li et al. [20] studied the impact-related properties of self-compacting concrete (SCC) with 0.5%, 0.75% and 1.0% steel fibers with an SHPB, and found that a steel fiber content of no more than 1.0% can be successfully used to prepare SCC with good workability and greatly improved impact resistance. Lok and Zhao [21] studied the impact response of SFRC through an SHPB and found that the unconfined uniaxial compressive strength of SFRC increases with strain rate in the same way as for plain concrete. Further, strain rate has a significant influence on the ductility of SFRC. At a high strain rate, the post-peak ductility is absent. Li et al. [22] studied the dynamic compressive behavior of SFRC at elevated temperatures by the SHPB and found that SFRC under dynamic compression at high temperatures displays strain rate sensitivity.

To fill the aforementioned knowledge gaps, this study focuses on the dynamic compression performance of SFRC by a 37 mm diameter SHPB system. Four steel fiber contents were incorporated into concrete, and samples were subjected into drying–wetting cycles for different corrosion durations. Through the experimental data, strain-rate effect, fiber reinforcement effect and the corrosion duration influence on the impact compression property of SFRC were identified. The dynamic increase factor results of these samples were compared with the existing models in previous published literature. An empirical dynamic increase factor profile characterization model considering fiber content, corrosion duration and strain rate is proposed.

## 2. Experimental Program

### 2.1. Raw Materials and Mixture Design

All tests were carried out using P.C. 32.5 Portland cement (Changzhou Longhua Co. Ltd. Changzhou, China) which meets the Chinese standard GB 175-2007 [23]. The chemical composition and physical properties of the cement are presented in Table 1. Natural river sand with a fineness modulus of 2.49 was used in this work as a fine aggregate, the absorption of the fine aggregate was 2.28%. The coarse aggregate used in this work was natural stone with a size of 5–10 mm, the absorption of the coarse aggregate was 1.3%. In this work, 4 different steel fiber volumetric content concrete samples were designed with a water-to-cement ratio of 0.56, and the details of mixture proportions are given in Table 2. The steel fiber used in this work was copper-plated steel fiber produced by China Changzhou Zhenping Engineering Fiber Co., Ltd. (Changzhou, China). The tensile strength and the Young’s modulus of the steel fibers were 600 MPa and 210 GPa, respectively, and the length of the fiber was 15 mm.

### 2.2. Sample Preparation

After mixing, all mixtures were cast into a cylinder mold with dimensions of 70 mm (diameter) and 140 mm (height), and placed in a room temperature condition at 23 ± 1 °C. After 24 h, the samples were de-molded and immersed in water at 23 ± 2 °C.

The dimensions of static compressive strength test cylinder samples were 70 mm × 140 mm. At present, there is no standard dimension requirement for compressive impact test samples. To minimize the frictional and inertial effects caused by the insufficient sample size, the following equation was suggested in reference [24]:

(1) $$\frac{l}{s}=\sqrt{\frac{3v}{4}}$$

where *l* is the thickness of the sample, *s* denotes the diameter of the sample, *v* represents the Poisson’s ratio of the sample, which can be taken as 0.3 for SFRC [25].

**Figure 1:**
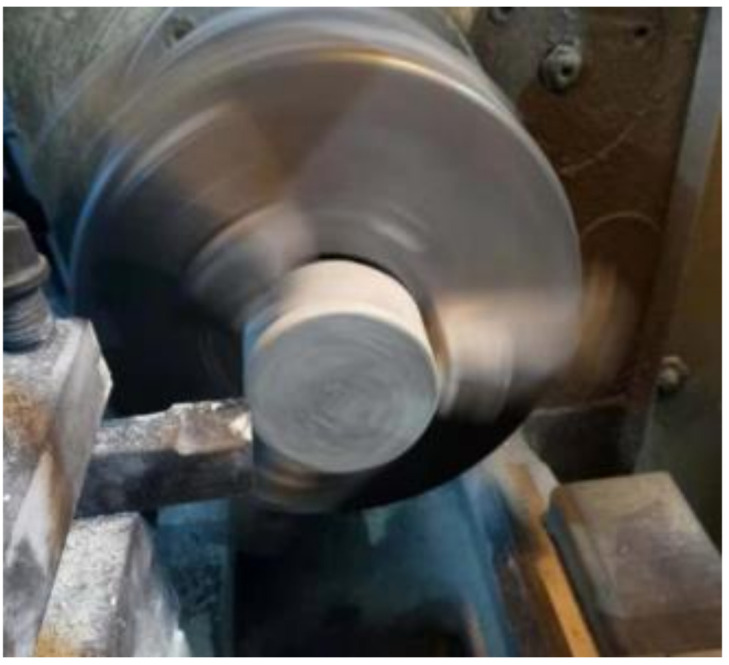
SHPB sample grinding.

Some other researchers [17,26] also proposed that the ratio between the thickness and the diameter of the cylinder compressive impact test sample should be between 0.5 and 5/3. Given the constant diameter of the cylinder sample in this work (i.e., 70 mm), the ratio between the thickness and diameter of the compressive impact cylinder sample was taken as 0.5, so the thickness of the impact test sample was 35 mm. The test samples for the split Hopkinson pressure bar (SHPB) were cut from the middle portion of the 70 mm × 140 mm cylinder sample and ground into the required thickness, as shown in Figure 1, and each mixture was prepared with 3 replicates.

### 2.3. Cyclic Drying–Wetting Accelerated Corrosion

**Figure 2:**
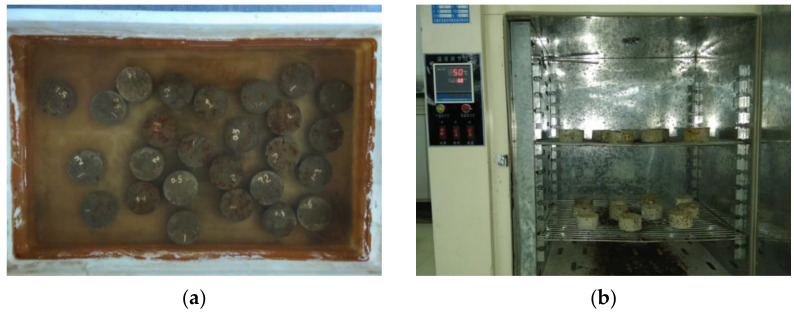
Drying–wetting accelerated corrosion samples. (**a**) Samples were immersed in NaCl solution, (**b**) samples were dried in an oven.

All samples for the static compressive strength test and SHPB impact test were cured in water for 28 days and then moved into cyclic drying–wetting accelerated corrosion conditions. Samples were immersed in 6% (by wt.) NaCl solution at a temperature of 23 ± 2 °C for 2 h (Figure 2a) and then dried in an oven at a temperature of 60 ± 1 °C for 10 h (Figure 2b), and every 12 h was a cycle. In this work, 4 different corrosion durations were selected (i.e., 0 days, 30 days, 60 days and 90 days).

### 2.4. 37 mm Diameter Split Hopkinson Pressure Bar (SHPB)

**Figure 3:**
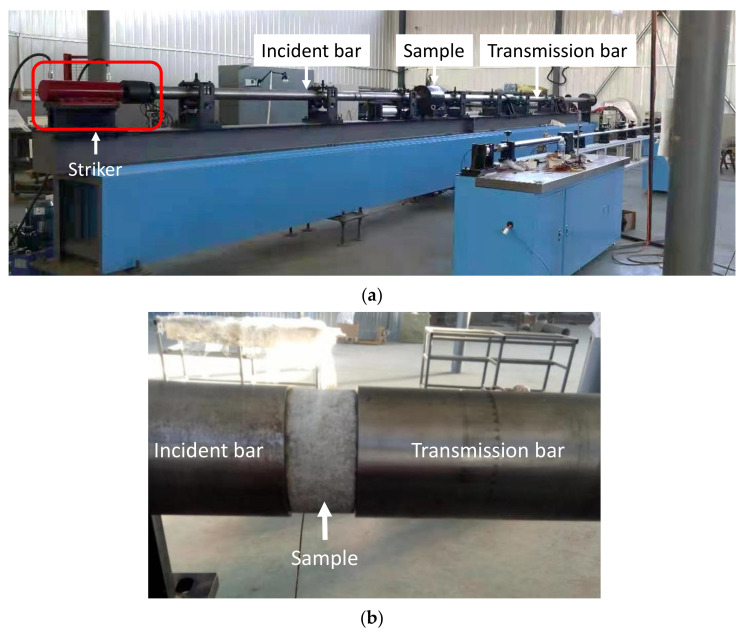
37 mm SHPB testing system. (**a**) SHPB testing apparatus, (**b**) sample was fixed in the bar.

A 37 mm diameter SHPB testing system is adopted in this work, as shown in Figure 3a. The system consists of a loading device, compression bar components and a data acquisition system. The loading device consists of a striker (bullet) and air cannon. The diameter and the length of the striker are 37 mm and 800 mm, respectively. The compression bar components include incident and transmission bars with a length of 2700 mm and 1800 mm, respectively. The diameter of the incident and transmission bars is 70 mm, as shown in Figure 3b. To ensure one-dimensional wave propagation and to facilitate large deformations in the sample when needed, the length-to-diameter ratio of incident and transmission bars should be at least 20 [27]. In this study, the ratios are 2700/70 = 38.6 and 1800/70 = 25.7 for incident and transmission bars, respectively. The data acquisition system consisted of strain and laser velocity observation subsystems. Two high-precision strain gauges are fixed on the incident and transmission bars. A signal amplifier is used in the subsystem to obtain reliable strain responses. The velocity observation system includes two sets of laser lights and a light-sensitive diode which can measure the speed of the striker during the test.

During the test, the striker was launched by a sudden release of the compressed air by the air cannon and accelerated in a long gun barrel until it impacted the end of the incident bar. In this study, 3 different striker velocities were selected, which would result in 3 different strain rates (*ε*) inside the sample (i.e., 20 s^−1^, 50 s^−1^ and 100 s^−1^). The stress wave was then generated by the impact of the striker on the incident bar. When the compression wave propagated into the interface between the incident bar and sample, part of the wave was reflected back to the incident bar and the rest of the wave was transmitted into the sample and reflected back and forth inside the sample. The stress level of the sample was gradually built up by these reflections and then compressed the sample.

## 3. Results and Discussion

### 3.1. Sample Damage

**Figure 4:**
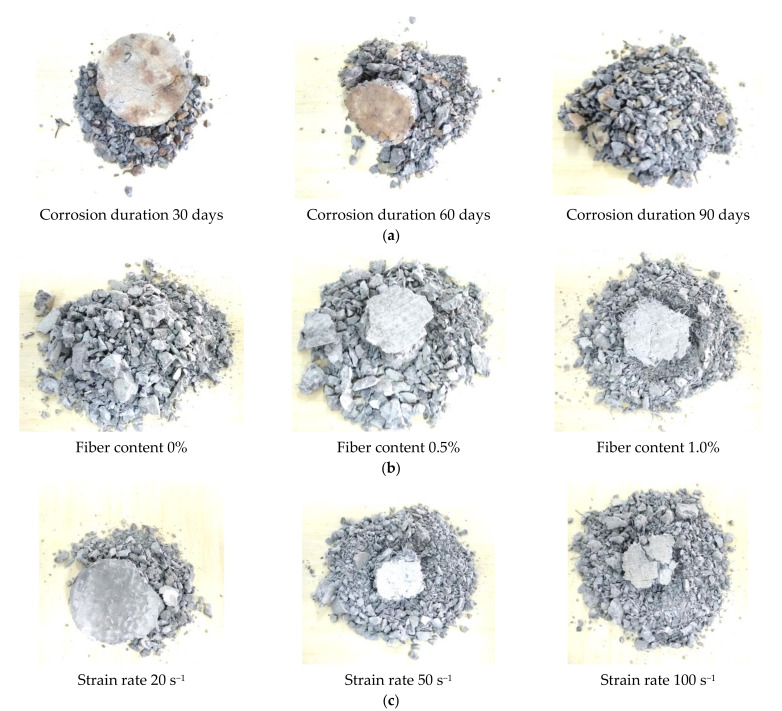
Fragmentation of the concrete samples, (**a**) strain rate = 20 s^−1^, steel fiber content = 1.0% under different corrosion durations. (**b**) strain rate = 20 s^−1^, corrosion duration = 0 days with different with different fiber content. (**c**) corrosion duration = 0 d, fiber content = 0.5% under different strain rate.

In each test, once the striker impacted the incident bar, the sample was crushed into several small fragments in a short time. Parts of the crushed samples are included in Figure 4. The general failure mode of the samples was fragmentation. It can be seen from Figure 4a that with the increasing of corrosion duration, the fragment sizes decreased, which indicates that under the same strain rate and fiber content, the longer corrosion duration decreased the dynamic impact resistance of the steel fiber samples. In Figure 4b, with the increasing of fiber content, the samples showed increased fragment sizes, and the plain sample (fiber content = 0%) showed completely broken fragments while the steel fiber-incorporated samples showed large fragments, which indicates that the SFRC has a higher dynamic compression performance. For the sample with the same corrosion duration and fiber content, a higher strain rate resulted in more fine fragments, as presented in Figure 4c, which is reasonable since the higher strain rate denotes a higher dynamic compression energy. The sample damage modes were very similar to the results reported by Hao and Hao [8].

### 3.2. Stress–Strain Curve of Tested Samples

**Figure 5:**
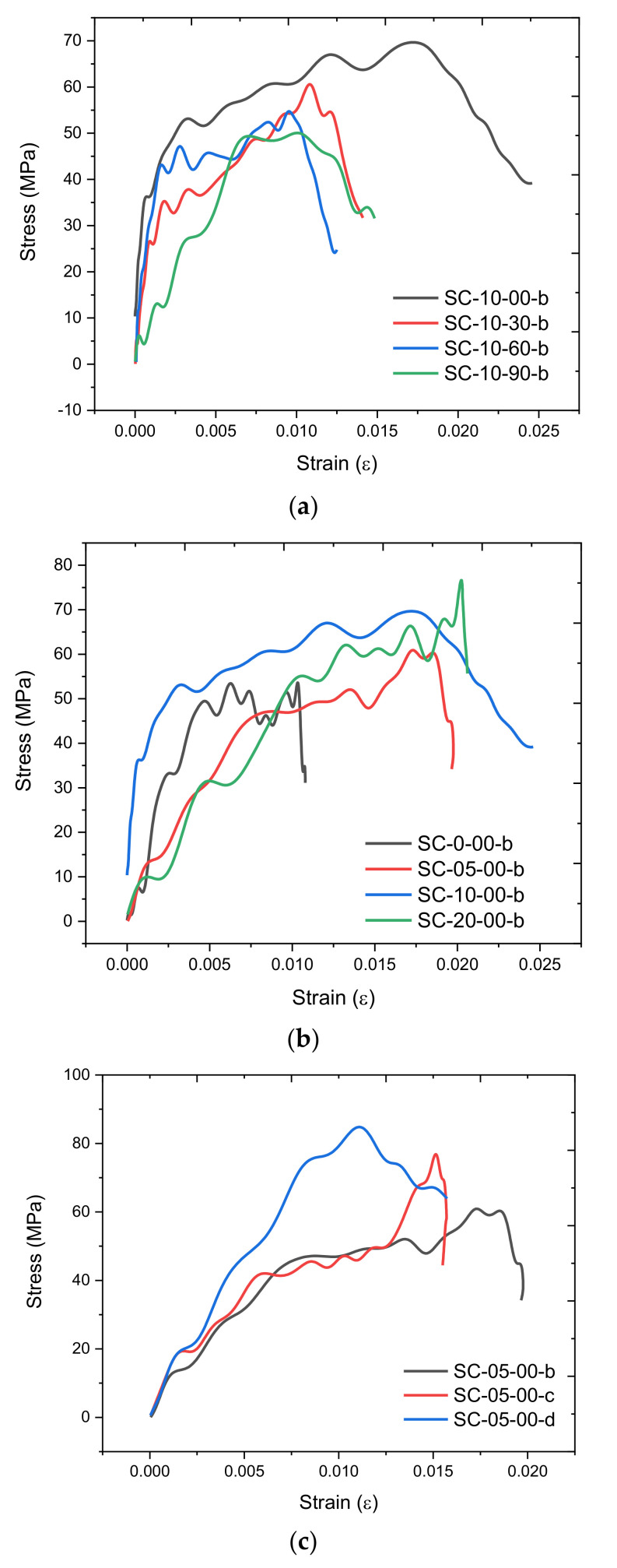
Stress–strain curves of concrete samples. (Note: sample was labeled as SC-XX-YY-Z, where XX denotes the fiber content, with 0, 05, 10 and 20 representing fiber contents of 0%, 0.5%, 1.0% and 2.0%, respectively. YY denotes the corrosion duration, with 00, 30, 60 and 90 denoting corrosion duration of 0 days, 30 days, 60 days and 90 days, respectively. Z denotes the strain rate level, with b, c and d representing strain level of 20 s^−1^, 50 s^−1^ and 100 s^−1^, respectively.) (**a**) Strain rate = 20 s^−1^, steel fiber content = 1.0% under different corrosion durations, (**b**) strain rate = 20 s^−1^, corrosion duration = 0 days with different fiber contents, (**c**) corrosion duration = 0 d, fiber content = 0.5% under different strain rates.

The stress–strain curves of all samples in Figure 4 are presented in Figure 5. The peak stress was defined as the dynamic strength of the sample. For the samples with the same strain rate and fiber content, the longer corrosion duration sample showed a decreased dynamic strength, and the corresponding strain was lower, as shown in Figure 5a, which shows that the increased corrosion duration would result in a poor dynamic compression performance of the steel fiber-incorporated concrete. Under the same strain rate and corrosion duration, the higher fiber content resulted in a higher dynamic strength and corresponding strain, as presented in Figure 5b, which implies that the incorporation of steel fiber increased the dynamic compression resistance of concrete. The influence of strain rate on concrete under the same corrosion duration and fiber content is included in Figure 5c. When strain rate increased from 20 s^−1^ to 100 s^−1^, the resulting strength increased, but the corresponding strain obviously decreased. The impact on samples happened in a short time (less than 1 s), and the higher strain rate denotes that more dynamic energy is transmitted into the sample in a shorter time, which would result in a higher dynamic strength, but the sample would break faster, so the corresponding strain would be lower.

### 3.3. Dynamic Increase Factor

The dynamic increase factor (*DIF*) was first proposed by Abrams [28] in 1917 and now is widely applied to analyze concrete performance under high-strain rate loads [17,29,30]. The *DIF* is normally defined as the ratio of the dynamic strength (*f_cd_*) to the static strength (*f_cs_*), as follows:

(2) $$DIF=\frac{f_{cd}}{f_{cs}}$$

**Figure 6:**
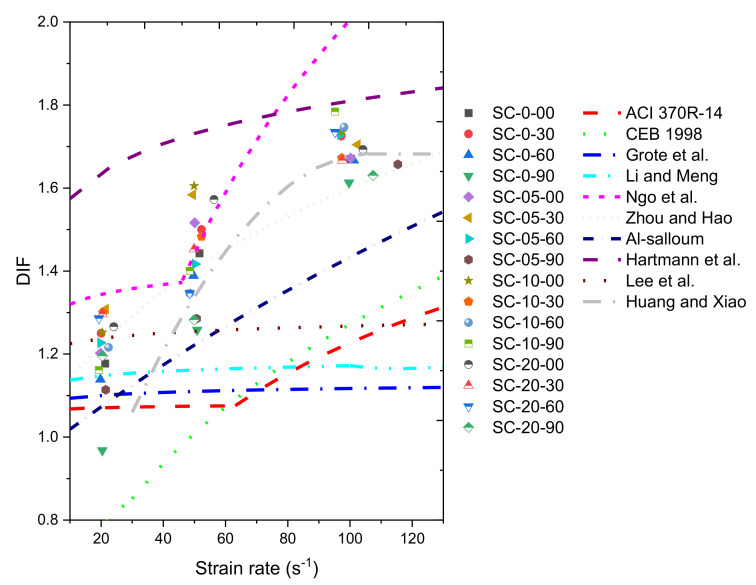
Experimental data in the present study and equations in the literature.

Many equations for *DIF* determination as a function of the strain rate were proposed based on the previously published experimental and analytical studies, as concluded in Table 3. Using the equations in Table 3, the distributions of *DIF* profiles suggested in previous literature and together with the experimental data in this study are included in Figure 6. Most of the equations can characterize the *DIF* profile in the 20 s^−1^ strain rate (i.e., Equations (3), (5)–(9) and (11)) and some equations agree with the *DIF* profile in the 50 s^−1^ strain rate (i.e., Equations (7), (8) and (12)) or 100 s^−1^ strain rate (i.e., (12)), while none of the equations can characterize these three strain rates.

**Table 3:** Equations in the literature.

References	Equations	
ACI 370R-14 [31]	$DIF=\left\{\begin{array}{cc} 0.00965\log\varepsilon +1.058 & \varepsilon \leq 63.1s^{-1}\\ 0.758\log\varepsilon -0.289 & \varepsilon >63.1s^{-1} \end{array}\right.$	(3)
CEB 1998 [32]	$DIF=\left\{\begin{array}{cc} {\left(\frac{\varepsilon }{3\times 10^{-5}}\right)}^{1.026\alpha } & \varepsilon \leq 30s^{-1}\\ \gamma_{s}{\left(\frac{\varepsilon }{3\times 10^{-5}}\right)}^{1/3} & \varepsilon >30s^{-1} \end{array}\right.$ $\mathrm{where}\;\alpha ={\left(5+\frac{9}{10}f_{cs}\right)}^{-1}$ $\;\mathrm{and}\;\gamma_{s}=10^{6.156\alpha -2}$	(4)
Grote et al. [33]	$DIF=\left\{\begin{array}{cc} 0.0235\log\varepsilon +1.07 & \varepsilon \leq 266s^{-1}\\ 0.882{\left(\log\varepsilon \right)}^{3}-4.48{\left(\log\varepsilon \right)}^{2}+7.22\log\varepsilon -2.64 & \varepsilon >266s^{-1} \end{array}\right.$	(5)
Li and Meng [34]	$DIF=\left\{\begin{array}{cc} 0.03438\left(3+\log\varepsilon \right)+1 & \varepsilon \leq 100s^{-1}\\ 1.729{\left(\log\varepsilon \right)}^{2}-7.137\log\varepsilon +8.530 & \varepsilon >100s^{-1} \end{array}\right.$	(6)
Ngo et al. [35]	$DIF=\left\{\begin{array}{cc} {\left(\frac{\varepsilon }{\varepsilon_{s}}\right)}^{1.026\alpha } & \varepsilon \leq \varepsilon_{1}\\ A_{1}\ln\left(\varepsilon \right)-A_{2} & \varepsilon >\varepsilon_{1} \end{array}\right.$ where *A*_1_ = 0.9866 − 0.0044*f_cs_*, *A*_2_ = 2.1396 − 0.0128*f_cs_*, *α* = 1/(20 + *f_cs_*/2) and *ε*_1_ = 0.0022*f_cs_^2^* − 0.1989*f_cs_* + 46.137	(7)
Zhou and Hao [36]	$DIF=\left\{\begin{array}{cc} 0.0225\log\varepsilon +1.12 & \varepsilon \leq 10s^{-1}\\ 0.2713{\left(\log\varepsilon \right)}^{2}-0.3563\log\varepsilon +1.2275 & \varepsilon >10s^{-1} \end{array}\right.$	(8)
Al-Salloum et al. [30]	$DIF=\left(3.54\varepsilon +430.6\right)/\left(\varepsilon +447.3\right)$	(9)
Hartmann et al. [37]	$DIF=0.5\varepsilon^{0.13}+0.90$	(10)
Lee et al. [38]	$DIF={\left(\frac{\varepsilon }{1\times 10^{-5}}\right)}^{0.0147}$	(11)
Huang and Xiao [17]	$DIF=\left\{\begin{array}{c} 0.002\varepsilon +1\\ -0.000103\varepsilon^{2}+0.02222\varepsilon +0.4859\\ 1.70 \end{array}\right.\begin{array}{c} \varepsilon \leq 30s^{-1}\\ 30<\varepsilon \leq 106s^{-1}\\ \varepsilon >106s^{-1} \end{array}$	(12)

**Figure 7:**
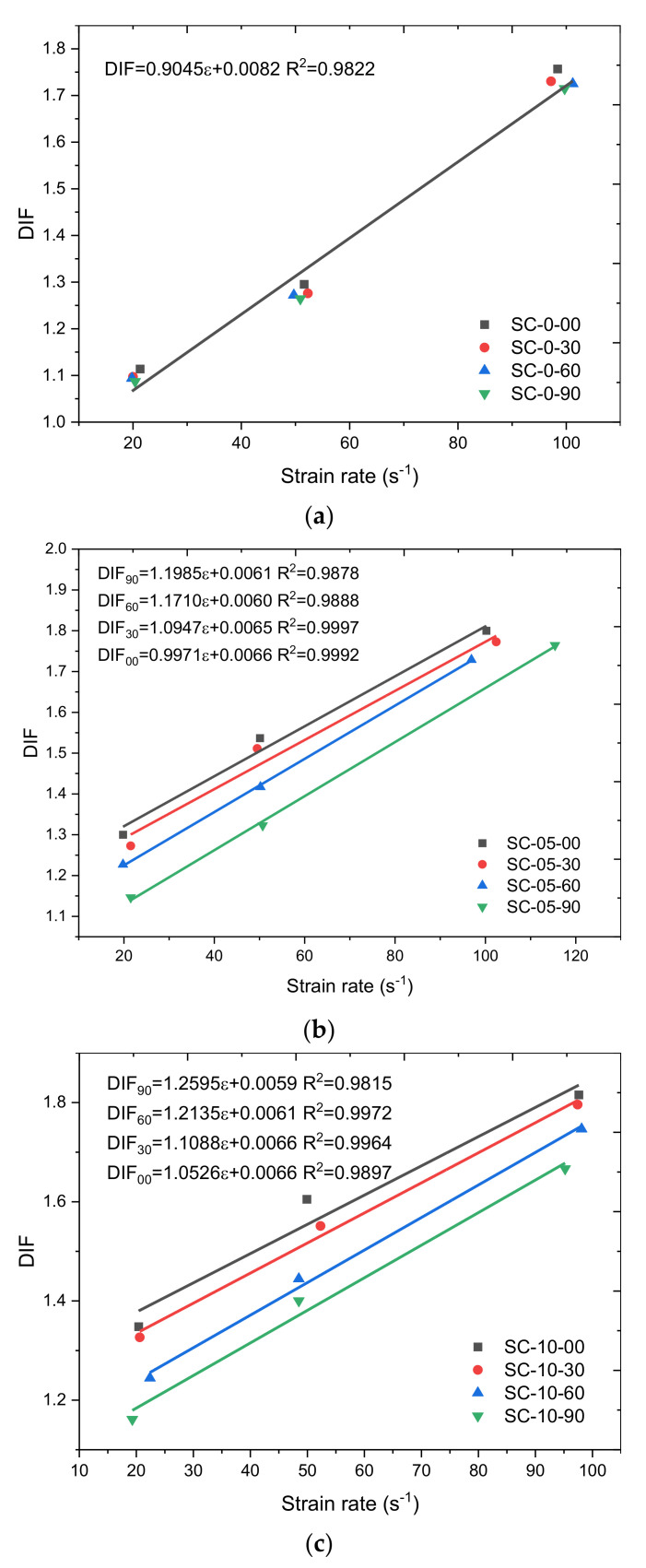

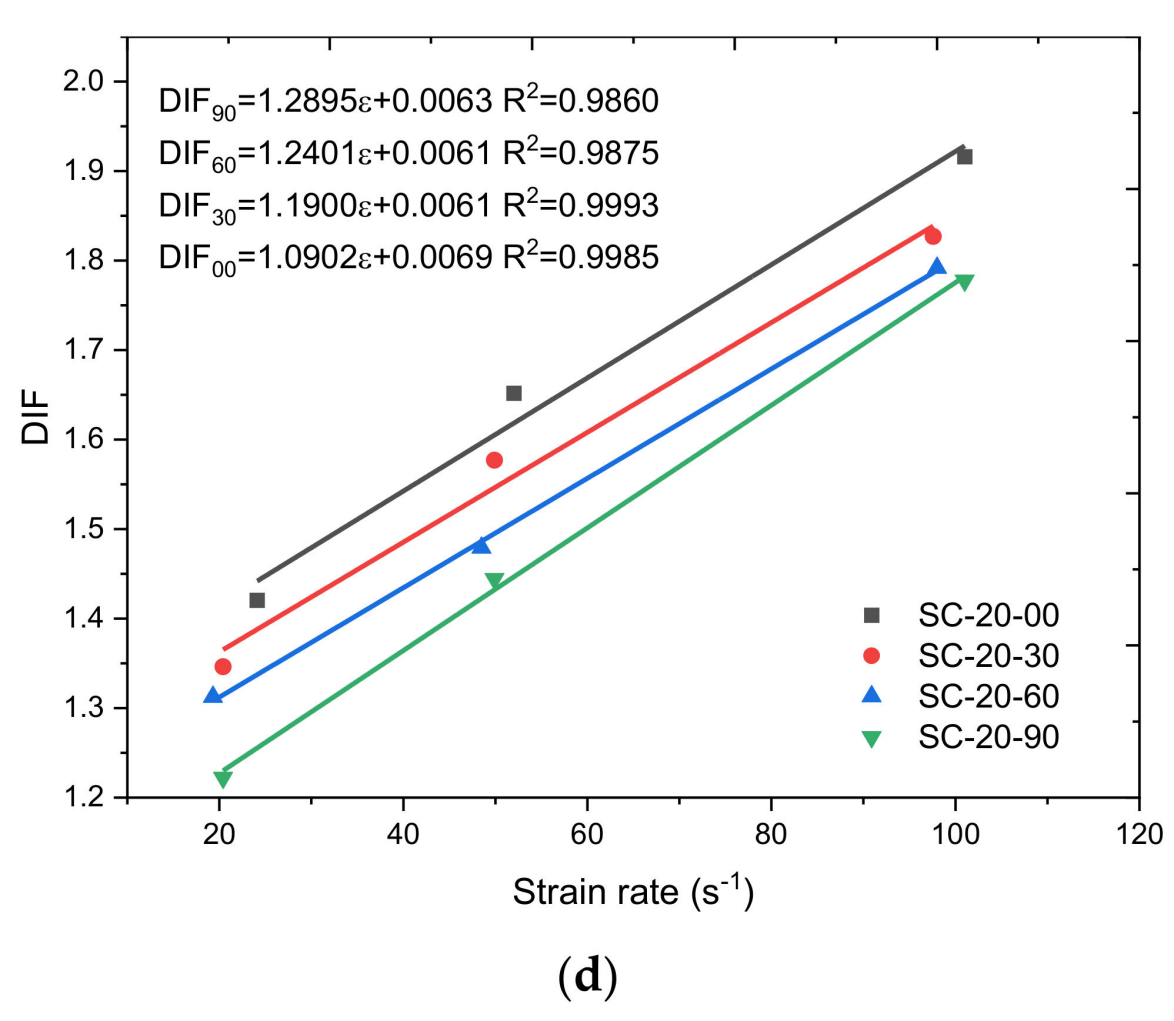
*DIF* profiles of steel fiber-incorporated concrete under different corrosion durations, (**a**) fiber content = 0%, (**b**) fiber content = 0.5%, (**c**) fiber content = 1%, (**d**) fiber content = 2%.

The *DIF* profiles of steel fiber-incorporated concrete under different corrosion durations are included in Figure 7. For plain concrete (Figure 7a), the *DIF* profile is independent of the corrosion duration since plain samples had been cured in water for 28 days, the cement had been well hydrated and the immersion in NaCl solution did not increase their dynamic resistance. The *DIF* profile of steel fiber-incorporated concrete decreased with the increasing of corrosion duration (Figure 7b–d) since the NaCl solution penetrated into the concrete through the micropore structures and induced corrosion of the steel fiber. The longer corrosion duration resulted in a higher corrosion degree, so the dynamic compression resistance decreased.

A series of linear relationships can be observed in Figure 7, and the fitting parameters R^2^ are all higher than 0.98, which implies that for steel fiber-incorporated concrete, in the 20 to 100 s^−1^ strain rate range, a linear relationship can be used to characterize the *DIF* profile well.

**Figure 8:**
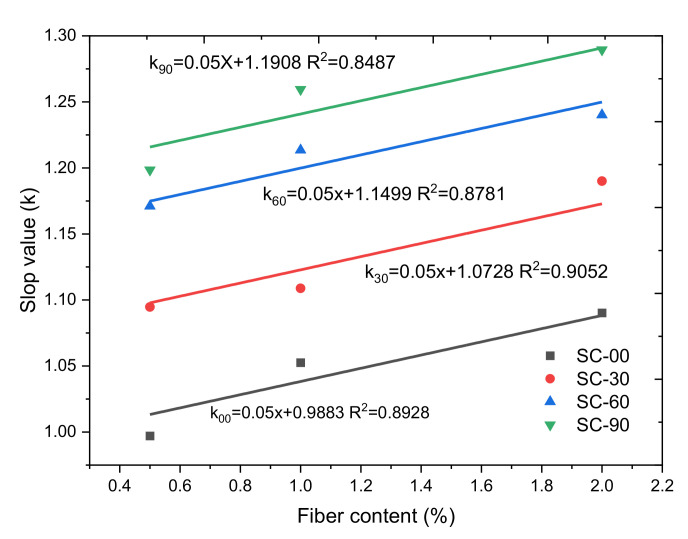
Slope values in terms of fiber content and corrosion duration.

**Figure 9:**
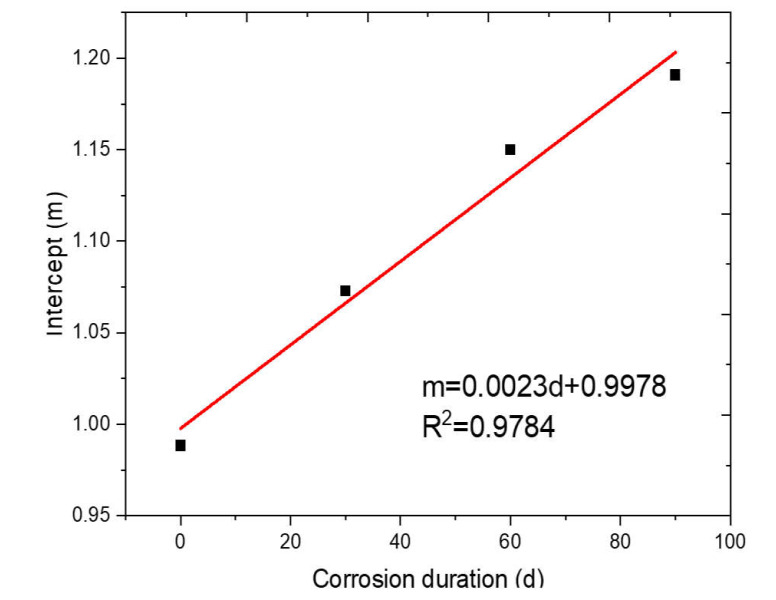
Fitting intercepts (*m*) in terms of corrosion duration (days).

For steel fiber-incorporated concrete, the slopes (*k*) and intercepts in Figure 7b–d linear fitting results are included in Table 4. The intercept values in Table 4 are independent of any parameter, and the average value of the intercept is 0.0063. The values of slope (*k*) show linear relationships with fiber content under different corrosion durations, as shown in Figure 8. The higher fiber content shows a higher slope value, which indicates that the higher dosage of steel fiber in concrete can contribute more dynamic resistance. The slope values in Table 4 represent the influence of fiber content on the strain rate increasing effect on the DIF profile. The strain rate increasing effect on the DIF profile is the same for concrete samples with the same fiber content. When the fiber content increases, the stain rate increasing effect on the DIF profile is increased due to the excellent dynamic mechanical properties of steel fiber. The linear fitting slope in Figure 8 was fixed at 0.05 for different corrosion durations. The linear fitting intercept (*m*) in Figure 8 shows a clear relationship with corrosion duration, as presented in Figure 9.

Thus, the combination of the fitting results in Figure 7, Figure 8 and Figure 9, the *DIF* profile for plain concrete (Equation (13)) and steel fiber-incorporated concrete with different fiber dosages under different corrosion durations (Equation (14)) can be depicted as:

(13) DIF=0.9045ε+0.0082

and

(14) $$DIF=\left(0.05x+0.0023d+0.9978\right)\varepsilon +0.0063$$

where *x* denotes the fiber dosage (%), *d* represents the corrosion duration.

It is worth noting that Equations (13) and (14) are proposed based on the experimental data in this work. For plain concrete, the application of Equation (13) is independent of sample age. For steel fiber-incorporated concrete, the application of Equation (14) should take the sample’s curing conditions into consideration where, in this case, the sample is immersed in water at 23 ± 2 °C for 28 days. The strain rate range in this work is limited to 20 to 100 s^−1^, other strain rates out of this range need to be studied further.

## 4. Conclusions

In this work, the dynamic compression performance of SFRC under different corrosion durations was studied by a 37 mm diameter split Hopkinson pressure bar (SHPB) system with various strain rates. The main conclusions can be drawn as follows:

1. The general failure mode of the SFRC under a high strain rate is fragmentation. The longer corrosion duration decreased the dynamic resistance of the SFRC. SFRC has a higher dynamic compression performance than plain concrete.
2. The longer corrosion duration results in a decreased dynamic strength and corresponding strain, and the higher fiber content results in a higher dynamic strength and corresponding strain. When the strain rate increases from 20 to 100 s^−1^, the resulting strength increased, but the corresponding strain decreased.
3. The previously proposed equations in the literature cannot precisely characterize the *DIF* profile for corroded SFRC. Two equations for plain concrete (Equation (13)) and corroded SFRC (Equation (14)) were proposed based on the experimental data in this work. The equations can be used to evaluate the plain and corroded SFRC dynamic compression resistance in the 20 to 100 s^−1^ strain rate range.

## Figures and Tables

**Table 1:** Chemical composition (% by mass) and fineness of the cement.

CaO	SiO_2_	Al_2_O_3_	Fe_2_O_3_	MgO	SO_3_	K_2_O	Na_2_O	LoI	Fineness (m^2^/kg)
64.47	20.87	4.87	3.69	2.13	2.52	0.65	0.11	0.77	368.9

**Table 2:** Mixture proportion for concrete.

Steel Fiber Content (%)	Steel Fiber (kg/m^3^)	Cement (kg/m^3^)	Fine Aggregate (kg/m^3^)	Coarse Aggregate (kg/m^3^)	Water (kg/m^3^)	Slump (mm)
0%	0	366	695	1134	205	210
0.5%	39	366	695	1134	205	180
1.0%	78	366	695	1134	205	170
2.0%	156	366	695	1134	205	150

Note: all aggregates were in saturated surface dry (SSD) condition.

**Table 4:** Linear fitting parameters.

Sample	Slope (*k*)	Intercept
SC-05-00	0.9971	0.0066
SC-05-30	1.0947	0.0065
SC-05-60	1.1710	0.0060
SC-05-90	1.1985	0.0061
SC-10-00	1.0526	0.0066
SC-10-30	1.1088	0.0066
SC-10-60	1.2135	0.0061
SC-10-90	1.2595	0.0059
SC-20-00	1.0902	0.0069
SC-10-30	1.1900	0.0061
SC-10-60	1.2401	0.0061
SC-10-90	1.2895	0.0063

## Data Availability

The data presented in this study are available on request from the corresponding author.
